# Prenatal affective cognitive training to reduce the risk of postpartum depression (PACT): study protocol for a randomized controlled trial

**DOI:** 10.1186/s13063-024-08316-1

**Published:** 2024-07-15

**Authors:** Anne J. Bjertrup, Jeanne Kofoed, Ida Egmose, Katrine Wendelboe, Victoria Southgate, Mette S. Væver, Kamilla W. Miskowiak

**Affiliations:** 1 The Neurocognition and Emotion in Affective Disorders Centre (NEAD), Psychiatric Centre Copenhagen, Mental Health Services in the Capital Region of Denmark, Frederiksberg, Denmark; 2 Department of Psychology, Faculty of Social Sciences, University of Copenhagen, Copenhagen, Denmark

**Keywords:** Randomized controlled trial, Postpartum depression, Affective cognitive training, Prevention, High-risk pregnancy, Cognitive bias modification, Attention bias modification, Emotion regulation, Working memory training, Mother-infant relations

## Abstract

**Background:**

Postpartum depression (PPD) affects 30–50% of women with a history of previous depression or bipolar disorder and 8% of women with no history of depression. Negative cognitive biases in the perception of infant cues and difficulties with emotion regulation are replicated risk factors. Current interventions focus on detecting and *treating* rather than *preventing* PPD. The aim of this randomized controlled intervention trial is therefore to investigate the potential prophylactic effects of prenatal affective cognitive training for pregnant women at heightened risk of PPD.

**Methods:**

The study will enrol a total of 292 pregnant women: 146 at high risk and 146 at low risk of PPD. Participants undergo comprehensive assessments of affective cognitive processing, clinical depressive symptoms, and complete questionnaires at baseline. Based on the responses, pregnant women will be categorized as either at high or low risk of PPD. High-risk participants will be randomized to either prenatal affective cognitive training (PACT) or care as usual (CAU) immediately after the baseline testing. The PACT intervention is based on emerging evidence for efficacy of affective cognitive training approaches in depression, including cognitive bias modification, attention bias modification, mindfulness-inspired emotion regulation exercises, and working memory training. Participants randomised to PACT will complete five individual computerised and virtual reality-based training sessions over 5 weeks. The primary outcome is the difference between intervention arms in the incidence of PPD, assessed with an interview 6 months after birth. We will also assess the severity of depressive symptoms, rated weekly online during the first 6 weeks postpartum.

**Discussion:**

The results will have implications for future early prophylactic interventions for pregnant women at heightened risk of PPD. If the PACT intervention reduces the incidence of PPD, it can become a feasible, non-invasive prophylactic strategy during pregnancy, with positive mental health implications for these women and their children.

**Trial registration:**

ClinicalTrials.gov NCT06046456 registered 21-09-2023, updated 08-07-2024.

**Supplementary Information:**

The online version contains supplementary material available at 10.1186/s13063-024-08316-1.

## Background and aim

Postpartum depression (PPD) is a severe mental illness with a prevalence of around 8% for women with no history of depression [1, 2] and 30–50% for women with a history of previous depression or bipolar disorder (mania and depression) [3–7]. Multiple factors are associated with risk of PPD, including a history of depression, unwanted pregnancy, low emotional support, low socio-economic status [8] pregnancy complications [4, 9–11], weak attachment feelings to the unborn child [12, 13], and emotion regulation difficulties [14]. Emerging evidence indicates that negative cognitive bias in the perception of infant cues and reactivity to infant distress plays a key role for the risk of PPD. For example, we showed that more negative ratings of infant crying during pregnancy were associated with a substantially increased risk of PPD with an odds ratio of 3.75 [15]. This finding is in line with evidence that negative cognitive bias is a risk and maintaining factor for depressive episodes [16, 17]. Pregnant women at known risk of PPD are commonly offered prophylactic *pharmacologic* interventions, most commonly with selective serotonin reuptake inhibitors (SSRIs) [18]. However, many pregnant women are reluctant to accept pharmacological interventions due to their potentially harmful effects on the foetus [19–21]. Psychological interventions, including talk therapy, psychoeducation, and midwife consultations, have been found to reduce PPD symptoms [22–26]. However, these interventions generally focus on *detecting and treating* rather than *preventing* PPD [27]. *Psychological preventive* strategies in pregnant women *before* PPD onset are thus urgently needed.

Mothers with PPD display more negative and/or flat facial expressions and fewer expressions signalling comfort, excitement, and happiness during mother-infant interactions [28–30] and show difficulties with regulating their own and their infant's emotions [31]. Further, more depressive symptoms have been associated with greater self-focus and lower levels of caregiving warmth in mothers [32]. Moreover, PPD may disrupt normal, rewarding behavioural synchrony between mother and infant, as evidenced by low levels of mutual gaze and affective touch between mothers with PPD and their infants [33]. At a neural level, mothers with PPD show reduced activation in response to infant stimuli in brain areas associated with reward, empathy, and emotion regulation including the orbitofrontal cortex, insula, and dorsal anterior cingulate cortex [34]. We found that mothers with remitted recurrent depression displayed more negative facial expressions when listening to infant cries, preferential visual attention toward sad (versus happy) infant faces and attentional avoidance of intense infant emotions on computerised tasks compared to never-depressed mothers [35]. Importantly, these negative affective cognitive biases were associated with *less maternal sensitivity* in mothers’ real-life interactions with their infants as well as lower scores for the infants on cognitive, communicative and motor development [35]. Another study found that negative cognitive responses to computerized infant stimuli mediated the link between maternal depression symptoms in pregnancy and adverse maternal behaviours toward their own infant one year after birth. These behaviours include intrusiveness, flat affect, and both covert (e.g. sarcasm) and overt (e.g. yelling) forms of hostility [36]. Such behaviours likely increase infant stress levels and withdrawal behaviour and hamper infants’ experience of positive emotions and their acquisition of emotion regulation abilities [37–39]. Aberrant affective cognition therefore seems to be a key treatment target to improve both mothers’ and their infants’ mental health.

Preliminary evidence points to the potential of several effective cognitive training approaches to reduce the risk of PPD. In a pilot study, cognitive bias modification (CBM) reduced negatively biased perceptions of infant distress in mothers with PPD [40]. No study has been conducted of computerised attentional bias modification (ABM) in mothers with or at risk of PPD, however, two studies of patients with depression found that two–three sessions of ABM were able to reduce negatively biased processing of emotional faces [41, 42]. A study of mindfulness-inspired emotion regulation exercises for mothers with PPD, anxiety or stress showed improvement in these mothers’ ability to remain calm and display empathic, well-regulated, and sensitive behaviour towards themselves and their infants [43]. Further, mindfulness was found to enhance adaptive vs. ruminative self-focused attention [44] and to increase precuneus-subgenual anterior cingulate cortex functional connectivity and amygdala–temporal pole functional connectivity, when mothers listened to infant cries [45]. Finally, there is emerging evidence indicating that enhancing working memory abilities through cognitive training results in better emotion regulation abilities across psychiatric diagnoses [46]. Such working memory training may have beneficial effects on mothers’ interaction with their infants because of the association between better emotion regulation abilities and less harsh parenting [47, 48].

### Aims and hypotheses

The aim of this randomized controlled trial (RCT) is to investigate the potential prophylactic effects of a short-term (5 weeks) prenatal affective cognitive training (PACT) intervention that combines computer and virtual reality-assisted techniques compared with care as usual (CAU) for pregnant women at heightened risk of postpartum depression (PPD). Furthermore, pregnant women who do not meet the inclusion criteria for the high-risk group, are included in the study and assessed at baseline during pregnancy and follow-up after birth. This allows us to explore the risk factors that predict PPD among all pregnant women. We hypothesize that (i) PACT reduces the incidence of PPD during the first 6 months postpartum, and the severity of depressive symptoms during the first 6 weeks postpartum and (ii) the effect of PACT on PPD onset and depressive symptoms after birth is mediated by change in affective cognitive responses to infant stimuli.

## Methods

### Proof of concept pilot study findings

There was no formal patient and public involvement in the trial design. However, insights and participant feedback obtained in our smaller-scale non-randomized proof of concept study [49] informed the current investigation. We designed, programmed, and piloted a short-term multimodal prenatal affective cognitive training (PACT) for pregnant women at heightened risk of PPD, based on the different lines of emerging evidence for affective cognitive training approaches. Results from the pilot study of two weeks training (four sessions) in 16 high-risk pregnant women indicated that the intervention is feasible [49]; 12 of 16 participants completed all assessments, they generally gave positive feedback and reasons for dropout were pregnancy-related limitations of physical mobility. Despite the small sample size, the study revealed a statistically significant treatment-related reduction in prenatal negatively biased perception of infant stimuli and negative emotional reactivity toward infant cry, which correlated with lower symptoms of depression six months after birth [49]. The intervention further improved sensitivity to infant expressions of happiness, “infant-directedness” in women’s facial expressions and visual attention toward infants, which we hypothesise may have positive effects on the interaction with their infant after birth [49]. The current PACT RCT builds on these feasibility and proof-of-concept findings.

### Study setting

The PACT trial is conducted at the Neurocognition and Emotion in Affective Disorders (NEAD) Centre at the Psychiatric Centre Copenhagen, Frederiksberg, and at the Department of Psychology, University of Copenhagen, Denmark.

### Recruitment and enrolment

**Fig. 1:**
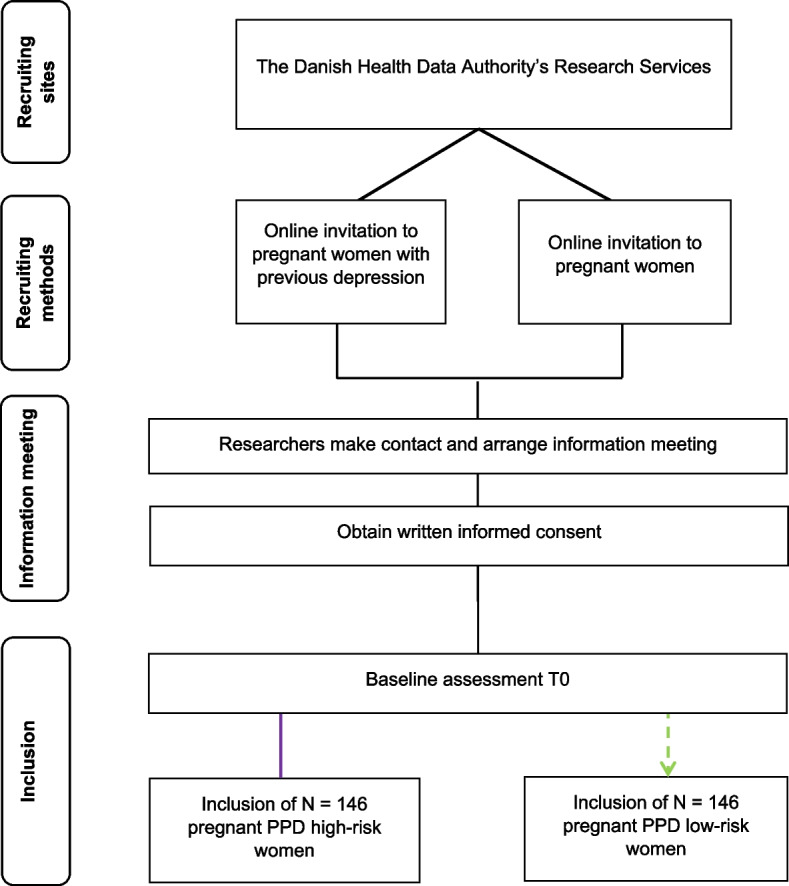
Recruiting sites, recruiting methods, and enrolment of participants. The purple lines refer to the enrolment of high-risk pregnant, and the green dotted lines refer to the enrolment of low-risk pregnant women

The trial will enrol a total of 292 pregnant women, including 146 women at a heightened risk of PPD and 146 women at a low risk of PPD. To identify eligible participants, we utilize the Danish Civil Registration (CPR) system to identify pregnant women (≥ 18 years of age) in their second or third trimester of pregnancy residing in the Capital Region of Denmark. The postdoctoral researcher on the study will invite potential participants via the online digital mailbox service used by 95% of the Danish population called “E-boks”. The invitation letter contains a link that interested participants can click to fill out their contact information and express their interest in an information meeting. For a visual representation of the recruitment process, please refer to Fig. 1.

### In- and exclusion criteria

General inclusion criteria are second or third trimester pregnancy, age ≥ 18 years, and ability to speak and read Danish. Inclusion criteria for the high-risk pregnant group are either negative cognitive bias in emotional reactivity to infant distress (cut-off ≥ 96 on a scale from 0 to 100) *or* high-risk status according to the Antenatal Risk Questionnaire (ANRQ). The ANRQ assesses specific key psycho-social risk factors that increase the risk of developing PPD and considers pregnant women at high risk of PPD if they a) have a personal history of severe mental illness, b) have experienced childhood emotional, physical, or sexual abuse or c) if their total score on psycho-social risk factors is above the cut-off (> 23). Pregnant women diagnosed with a personality disorder will be excluded from randomization due to their higher treatment resistance [50]. However, they will still be included at baseline and follow-up after birth to explore the risk factors that predict PPD. Inclusion criteria for the low-risk pregnant group are the absence of a personal or family history of mental illness, the absence of negative bias and three or less of the additional risk factors mentioned above. General exclusion criteria are schizophrenia and current substance use disorder. Additionally, participants who have a score of 9 or more on the Hamilton Depression Rating Scale-6 items (HDRS-6), indicating moderate depression [51], will be postponed or excluded. This criterion is crucial for our prevention-focused approach, ensuring participants without active depression, allowing us to prioritize prevention over treatment.

### Statistical power calculation

In a previous study from our group on affective cognition in high-risk pregnant women, 50% went on to develop PPD [15]. On average, these women rated infant cries 0.47 standard deviations *more negatively* than high-risk women who remained healthy. Hence, 83% of high-risk women who went on to develop PPD, exhibited a negative bias in their responses to infant cries during pregnancy compared with 48% high-risk women who remained healthy. Therefore, targeting negative affective cognitive response to infant emotion may reduce the risk of PPD. Based on this PPD incidence of 50% [15], we aim to demonstrate a clinically significant reduction of the incidence of PPD from 50 to 25% (i.e. reducing the risk to half). To reach a statistical power of ≥ 80% for detecting this effect at a two-sided 0.05 alpha level, we need a sample of *n* = 58 pregnant high-risk women with complete data per intervention arm (https://clincalc.com/stats/samplesize.com). To accommodate for an expected dropout rate of up to 20% (as in our pilot study), we aim to include *n* = 73 women per treatment arm, i.e. *N* = 146 trial participants in total.

### Randomization

Pregnant PPD high-risk women are randomized to PACT or care as usual (CAU) using the REDCap randomization module. We stratify randomization with age (above/below 30 years) and parity (primiparous v. multiparous). A researcher outside the group generated the allocation sequence by creating an allocation table with allocation in blocks of varying size using Sealed Envelope Ltd [52]. This allocation table is used by REDCap’s randomization module. The postdoc on the project conducts the randomization. Allocation concealment will be ensured as REDCap will not release the randomization code until the patient has been recruited into the trial and the “randomize button” in REDcap has been selected.

### Study design

**Fig. 2:**
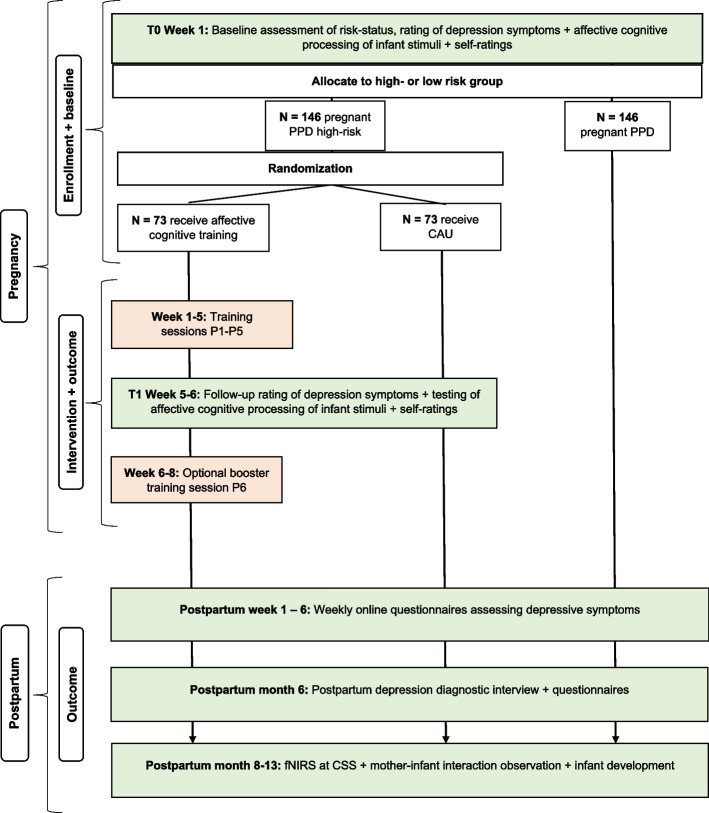
Flow-chart of the study design

**Fig. 3:**
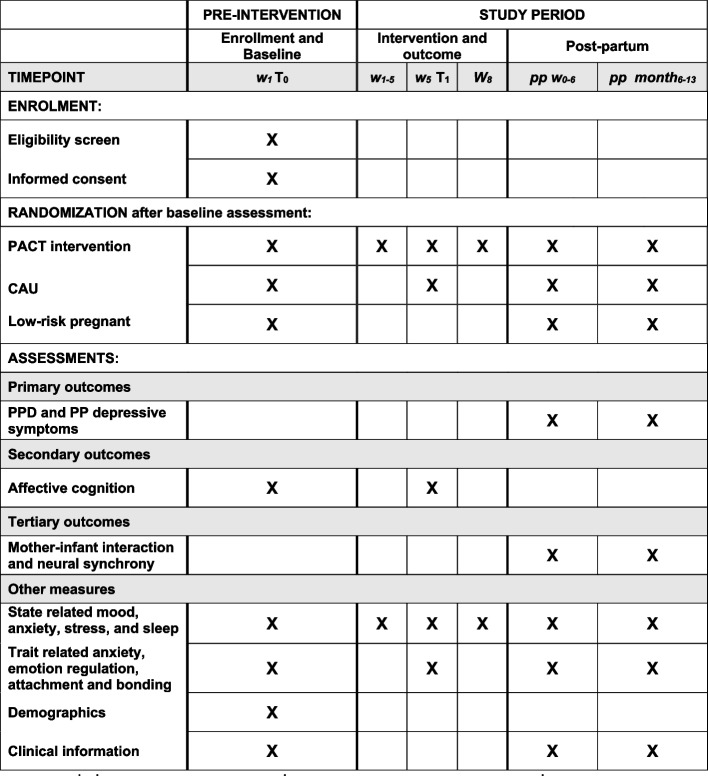
Schedule of enrollment, interventions, and assessments

The trial is a parallel-group, outcome-assessor-blind, superiority randomized controlled study with a 1:1 allocation ratio. See Fig. 2 for a flow chart of the study design. To ensure the transparency and completeness of the study design and conduct, we followed the Standard Protocol Items: Recommendations for Interventional Trials (SPIRIT) 2013 statement and included a SPIRIT figure and checklist (see Fig. 3 and Additional file 1: Table S1).

### Procedure

At baseline (T0), all pregnant women undergo a comprehensive (2-h) assessment of affective cognitive processing of infant stimuli and non-emotional cognition, clinical rating of depressive symptoms, and completion of questionnaires regarding attachment to the unborn child, bonding to own parents, emotional relationship with their partner, demographics, clinical history, and state- and trait anxiety (details below). Further, a hair sample is collected from women to assess cortisol levels as a biological measure of stress. Based on the responses on questionnaires and affective cognitive processing, pregnant women are categorised as either at high or low risk of developing PPD (see the “In- and exclusion criteria” section above for specific inclusion criteria for high- and low-risk groups, respectively). High-risk participants are randomized to either PACT or CAU immediately after the baseline testing. Participants in the active intervention group complete a total of five individual training sessions over a period of approximately 5 weeks (for details on the study design, see Fig. 2). Around 5-6 weeks after the baseline assessment, all high-risk pregnant women (in the PACT or CAU arms) undergo their second assessment (T1) of affective cognition and self-ratings, including attachment feelings to the unborn child. For women receiving PACT, this assessment takes place within 1 week after the fifth training session. Lastly, if they have not given birth yet, women in the intervention group are offered a *booster* training session approximately 6-8 weeks after their baseline assessment. If participants face challenges attending in-person sessions, for example due to the late stage of their pregnancy, we will offer to conduct two sessions over the telephone or an online meeting. While computer training will not be feasible in this format, we will emphasize the key points from previous sessions and guide them on how to maintain their progress through home-based exercises like mindfulness techniques.

One week after their due dates, participants receive an e-mail with a link to 5-min online questionnaires regarding the birth, the infant’s health and well-being and mothers’ potential experience of depressive symptoms. On a weekly basis during the first 6 weeks after giving birth, mothers receive links to an online questionnaire to assess their depressive symptoms.

Six months after birth, we assess the presence or absence of PPD with a diagnostic interview conducted in person or over the telephone. Mothers also fill out questionnaires regarding their emotional reactions when their infants cry, postpartum bonding, parental reflective functioning, parental stress, and infant development. We assess if they have used any of the strategies learned during the PACT intervention in pregnancy. Around 8–13 months after birth, we will invite mothers and their children to visit the Department of Psychology for a functional near-infrared spectroscopy (fNIRS), where brain activity in both the child and mother is measured simultaneously during an interaction. From this, we assess interpersonal neural synchrony, which is the correlation of the mother’s and child’s brain activity over time during social contact [52]. We will make a short video recording of the mother-infant interaction, which will be coded by researchers blind to group status. Finally, at this visit, we assess child development. The researchers conducting follow-up assessments are blind to group status.

### Intervention

The research group developed the PACT intervention, which consists of seven computerized and VR-based affective cognitive training tasks to provide an immersive and interactive experience. During each 1-h training session, participants engage in affective cognitive training tasks and reflect on how the practices and strategies learned during the training sessions can be effectively applied in their daily lives. The therapist will guide the participant in exploring various ways to integrate these techniques into their routine and adapt them to real-life situations.

In the first session, participants receive a detailed introduction to affective cognitive training, gaining an understanding of how and why affective cognitive processes are targeted. Participants' motivations and goals for the intervention are also discussed, and they receive a personal notebook to record their objectives. Throughout the following sessions, participants reflect on the practical application of each training task in their daily lives and document their experiences and progress in the notebook. The purpose of each computerized task is explained, its relevance to participants’ goals is discussed, and assistance is provided in translating the learned concepts to everyday life. In the final session, participants receive a leaflet containing information and exercises from the training sessions, discuss their benefits from the intervention, and are encouraged to continue practising what they have learned. The five training sessions are held over a period of approximately five weeks.

At the beginning of each session, participants select one of four different mindfulness VR scenarios lasting 5 to 10 min: a desert, a forest, the northern lights, or a beach by the sea. After the mindfulness exercise, participants complete an attentional bias modification (ABM) task and then proceed to the third task, which involves responding to infant videos with sensitive infant-directed facial expressions. The fourth task focuses on implicit emotion regulation training to enhance cognitive control abilities. Subsequently, participants engage in a cognitive bias modification (CBM) task that modifies negative cognitive biases in the perception of infants’ expressions. The sixth task involves explicit emotion regulation using mindfulness strategies and exposure to virtual reality scenarios, while the intervention concludes with a mindfulness-based visualization task.

The ABM, infant-directed facial expressions, and VR tasks utilize "biofeedback" technology, which employs real-time eye-tracking, facial expression analysis, and gaze analysis, respectively, to monitor participants’ responses to emotional infant signals. Continuous feedback is provided based on these responses for an effective and immersive training experience.

Attentional bias modification: In the ABM task, they watch infant videos on a laptop. Continuous information from real-time eye-tracking feeds back into the task and renders the video either blurred and red or clear depending on whether women look away or fixate on infant faces, respectively. This biofeedback directs participants’ attention toward infants’ faces. By repeatedly reinforcing attentional bias towards infant cues, this task aims to modify attentional biases away from intense infant emotion and promote more attentive responses to infant emotional stimuli.

Facial expression training: During the infant-directed facial expression task, women watch infant videos and are instructed to mirror infants’ emotional states with their facial expressions. An algorithm (Affectiva Affdex [53]) within the iMotions software continuously detects the participants’ facial expressions from facial action units (AUs), which are constantly estimated against predefined criteria for sensitive mirroring of specific infant emotions in real-life mother-infant interactions [54]. Participants receive feedback and modify facial expressions accordingly. This training enhances participants' reflection on the importance of facial expressions and communication with their infants, fostering a deeper understanding of the role facial expressions play in mother-infant interactions and promoting more effective non-verbal communication.

Cognitive and emotional control training: The cognitive control training task is a verbal letter-variant* N*-back task, with distracting infant cry sounds coming and going. Participants are instructed to indicate when a letter presented on the screen matches the one displayed *N* steps back by pressing a key. Participants are instructed to focus on the task even when infant cry sounds appear. By training participants to sustain attention and inhibit distractions, this task strengthens their cognitive control abilities, which are crucial for effective emotion regulation.

Cognitive bias modification: In the CBM task, women watch infant emotional face images each presented for 2 s. and are asked to evaluate, as fast as possible, whether the infant is happy or distressed after which they receive feedback corresponding to the emotion displayed. For 10 ambiguous infant faces, participants are continuously told that the infant is happy, which lowers the threshold for perceiving infant happiness.

Virtual reality-based emotion regulation training: Virtual reality-based emotion regulation training incorporates exposure to being with an infant, allowing participants to gradually acclimate to the presence of infants in a controlled environment. The VR scenario presents a playroom-like environment with various toys. In the VR scenario, participants are immersed in situations where infants are crying, laughing, or relaxing, with one infant present at a time. At the beginning of each VR scenario, participants are instructed by a voice-over and provided with a specific emotion-regulation strategy that focuses on their attention and focus. They are instructed to maintain visual contact with the infants’ faces while applying this strategy. To reinforce attention towards the infant, a “circle” within the VR scenario guides participants' attention back to the infant if they gaze away. By practising this task, participants improve their emotion regulation skills by learning methods to remain calm, direct their attention towards the infants rather than their own emotions, and consider the needs of the infants. This immersive virtual reality experience provides a safe and controlled environment for participants to develop and enhance their ability to regulate their emotions effectively in real-life interactions with infants.

Visualization of the baby: The visualization task involves a short meditation, where participants are instructed to breathe, close their eyes, visualize themselves with their baby, and send loving, kind, and warm thoughts and feelings toward the baby in their tummy. Through engaging in the visualization exercise, participants can cultivate a positive emotional connection with their unborn child, fostering feelings of care and bonding, and ultimately reducing depression by providing a sense of emotional well-being during pregnancy.

Care as usual: Participants receiving CAU will receive the standard care provided to pregnant women without any additional experimental interventions. It typically involves routine prenatal care, which may include regular check-ups, medical assessments, and general support from healthcare professionals. In some cases, high-risk pregnant women with a history of severe mental illness may receive additional specialized care, such as more consultations with midwives or the consideration of psychotropic medication, to address their specific needs and ensure their well-being throughout pregnancy.

### Materials

Computerized testing and training are run on a Lenovo IdeaPad Gaming 3 15.6" laptop using E-prime version 2.0 and iMotions Software version 9.0 and integrated biosensor hardware (iMotions A/S, Copenhagen, Denmark) including eye-tracking (Smart Eye AI-X) [55–57], galvanic skin responses (GSR) and facial expression analyses [53]. Training and testing VR scenarios are created in collaboration with the VR company Khora. Specifically, eight 4–9-month-old infants were video filmed with an Insta Titan camera, recordings were edited in Premiere Pro and VR scenarios programmed in Unity software and were run on an HTC Vive 2 Eye VR headset for baseline tests and a PICO VR headset for training sessions. The interpersonal synchrony of brain activity between mother and child is measured using fNIRS with the Artinis system, which includes BabyBrite, PortaSync hardware, and OxySoft software.

#### Clinical assessments, interviews, and questionnaires

At baseline T0, participants fill out questionnaires regarding their stress levels, sleep, relation to own parents, unborn child and partner, and depressive symptoms with the State and Trait anxiety questionnaire (STAI-S and T) [58], Pittsburgh Sleep Quality Index [59], Perceived stress [60], Parental Bonding Inventory [61], Maternal Antenatal Attachment Scale [62], Experiences in Close Relationships, Cognitive Emotion Regulation Questionnaire [63] and Edinburgh Postnatal Depression Scale (EPDS) [73, 74]. The EPDS is the internationally most widely used screening tool for PPD and in the Danish population, a cut-off of 11 or higher is the most indicative when screening for PPD [64]. They also rate their experience in VR with the Multimodal Presence Scale [65]. As mentioned previously, participants also fill out the ANRQ [66], which is used to determine their risk status for PPD.

At baseline, participants are interviewed with the HDRS-6 [51, 67], the Standardized Assessment of Personality – Abbreviated Scale (SAPAS) [68] and assessed with the Screen for Cognitive Impairment in Psychiatry (SCIP) [69].

Six months postpartum, mothers undergo an interview utilizing the Present State Examination (PSE), a component of the Schedules for Clinical Assessment in Neuropsychiatry (SCAN) [70], to assess the presence of depressive episodes since childbirth. The clinicians conducting the diagnostic interviews have received specific training and ongoing supervision from the project leader to ensure valid and reliable assessments.

Further, mothers rate their emotions when their child cries with the My Emotions Questionnaire [68]; they fill out the Postpartum Bonding Questionnaire [69] and Parental Stress Scale [70] and evaluate their child’s development with Ages-and Stages Questionnaire: Socio-Emotional (ASQ:SE) [71]; and their reflective functioning is assessed with Parental Reflective Functioning Questionnaire (PRFQ-1) [72].

Mother-infant interaction videos from 8 to 13 months after birth follow-up will be coded with the manual-based tool Coding Interactive Behaviour (CIB) [71] and infant development will be assessed with the Bayley Scales of Infant and Toddler Development 4th edition [72, 73]. Both assessments are conducted by trained researchers blind to group status.

#### Data entry, coding, security, and storage

All data entry for this trial will be conducted directly into REDCap, a secure web application designed for research data capture. REDCap ensures high data quality through several mechanisms: field validation, e.g. ensures that dates are correctly formatted and within specified ranges, and that numerical values fall within predetermined limits. Essential fields are marked as required to prevent missing data, for example when participants fill out questionnaires. REDCap is hosted on a secure server. Access to the system is restricted to authorized personnel through unique user credentials, ensuring data confidentiality and integrity. All data will be collected electronically via REDCap by trained research staff, ensuring consistency and accuracy, and eliminating the need for paper-based Case Report Forms (CRFs).

The study is conducted in compliance with existing laws on data protection. Any changes to the protocol will be submitted for approval to the Ethics Committee. Deviations from the protocol will be documented, and the protocol will be updated in the clinical trial registry as necessary. To ensure confidentiality, all data collected during the course of the research will be kept strictly confidential, pseudonymized and accessed only by members of the trial team. Participants will be allocated a pseudonymous ID number to protect their identities. Data from interviews, ratings, questionnaires, and cognitive tests are in REDCap, a secure web application for research data capture. Computerized test data are also stored securely on a server within the Mental Health Services, Capital Region of Denmark. In the current trial, we are collecting hair samples from participants for hair cortisol analyses. These samples will be analysed in one batch after all data collection is completed. The hair samples are kept in envelopes labelled with the participant's ID number and the date of collection. The envelopes are stored securely in a locked cabinet within a locked office. The hair samples will not be kept for future ancillary studies. The de-identified data will be securely stored for ten years after data collection has ended and will then be deleted.

### Outcome measures

The *primary outcome* is the difference between mothers receiving PACT versus CAU in (i) the incidence of PPD during the first six months after birth, assessed with the Present State Examination (PSE), which is a part of Schedules for Clinical Assessment in Neuropsychiatry (SCAN) [70].

The *three secondary outcomes* are differences between participants receiving PACT and CAU in (i) severity of depressive symptoms during the first six weeks after birth, (ii) the reduction in negatively biased cognitive processing of infant stimuli from baseline to follow-up during pregnancy (T1), and (iii) the self-rated parental stress six months after birth.

*Tertiary outcomes* involve assessing differences in infant development, mother-infant interaction measures (e.g. sensitivity, intrusiveness, dyadic reciprocity), and changes in facial expressions and visual attention towards infant stimuli (during pregnancy) between the PACT and CAU groups.

### Statistical analyses

Group differences in the prevalence of PPD will be analysed with generalized mixed model and linear mixed models, respectively. Group differences in affective cognitive changes from baseline to follow-up (in pregnancy) will be analysed with linear mixed models. We will apply appropriate imputation methods before fitting the model to address missing data concerns. With mediation analyses, we will investigate if depressive symptoms and the prevalence of PPD after birth are mediated by changes in affective cognition. Associations between affective cognitive performance on training tasks and changes in affective cognitive outcomes and PPD prevalence will be explored with regression analyses. All analyses will involve adjustment for variables on which participants may differ at baseline, including socio-demographic factors, attachment feelings to the unborn child, child gender, clinical history, and depressive symptoms during pregnancy. We will use the SPIRIT reporting guidelines [74].

### Feasibility

The goal is to recruit 146 high-risk and 146 low-risk pregnant participants through invitations sent to the women’s digital mailboxes. However, the possibility of non-response bias and dropout exists due to varying levels of participant inclination or ability to participate. To reduce this potential bias, multiple strategies are implemented, such as reminders, and varied phrasings in the invitation emphasizing the investigation's importance to different degrees. The impact of each strategy may vary depending on population characteristics and recruitment situations [75]. After completion of recruitment, post hoc analysis will be conducted to compare response rates and bias across various sociodemographic groups to determine the extent of any potential non-response bias or dropout in the study. The recruitment process will continue until the target sample size is achieved.

### Compensation to participants

Participants in the intervention, CAU and low-risk control groups receive a gift worth 400 DKK, 200 DKK, and 150 DKK, respectively as an acknowledgement and compensation for their time and effort invested in the project. They receive the gift at the final assessment during pregnancy. If participants choose to withdraw from the study before completion, they receive the gift at their final visit. Participants can get compensated for their time off work (100 DKK/hour) for the outcome assessments if they show documentation of lost income. Transportation costs can be refunded corresponding to the costs of public transportation from the participant’s address of residence to Department of Psychology, Copenhagen. This complies with existing requirements regarding compensation.

### Research ethics

The possible risks related to participating in this study, include experiencing motion sickness in VR, or fatigue due to task repetitions. Further, the affective cognitive tasks are challenging and may be overwhelming to some participants, which could lead to negative self-perceptions regarding task performance and possibly motherhood. We acknowledge such feelings and seek to reduce them by underscoring that the tasks are generally very challenging and that their effort is valuable. In general, the potential risks related to study participation are not associated with persistent harmful consequences for participants’ physical or mental health. In case mothers indicate that they have thoughts of self-harm and experience severe depressive symptoms (with Edinburgh Postnatal Depression Scale (EPDS) ≥ 11 [64, 76]), the named postdoc researcher and psychologist on the project will contact them and, if needed, refer them to psychiatric treatment. Therefore, we consider the potential risks justifiable and the study responsible. Moreover, the potential benefits for the high-risk pregnant women in the active arm are that they may have a lower risk of PPD, and they get support and attention from the psychologist during the study. All participants are followed up after birth and contacted and guided if severe depression symptoms or self-harm arise. The results of the study will have important implications for the scientific understanding of and clinical work with pregnant women at high risk of PPD. Therefore, we argue that the benefits of the study outweigh the potential risks.

### Dissemination of results

The project will result in four articles published in peer-reviewed international scientific journals. The first article will report primary outcome findings, i.e. the effect of the intervention on PPD onset. The second article will involve secondary outcomes, including affective cognitive changes, and how these mediated effects on depressive symptoms or PPD. The remaining two articles report exploratory outcomes, including how affective cognitive performance on training tasks correlates with changes in affective cognitive outcome measures and PPD prevalence at follow-ups.

### Project status

Version 1 of the study protocol was submitted on October 23, 2023. The study was initiated in January 2023 and when the protocol was submitted, the study included 90 high-risk and 60 low-risk participants. At the revision in June 2024, 130 high-risk and 130 low-risk participants have been tested at baseline. The recruitment and treatment phase of the project is planned to take place from January 2023 to December 2024. The final follow-up is expected to be completed in July 2025.

### Oversight and monitoring

In this relatively small-scale and low-risk study, which does not involve testing a medical device, a trial steering committee was not established nor was the establishment of a Data Monitoring Committee (DMC) required. Given the relatively low-risk nature of the intervention, we decided to employ no specific stopping rules or interim analysis. The trial will proceed as planned unless unforeseen issues arise, in which case the Ethics Committee will be promptly informed, and appropriate actions will be taken to ensure participant safety.

The sponsor is an independent research fund managed by the Mental Health Services in the Capital Region of Denmark. This Research Fund had no role in the design or conduct of the study.

## Discussion

During this trial, we made several modifications to the intervention and sample size to enhance feasibility and effectiveness. These changes have only been conducted after ethical approval from the Committee on Health Research Ethics in the Capital Region of Denmark. Early in the study (December 2023), we reduced the number of intervention sessions from six to five. Some participants, residing far from the research site and nearing their due dates, faced physical limitations in travelling. To accommodate them, we allowed some sessions to be conducted online or via telephone, provided they attended at least three sessions in person. Additionally, booster sessions were made voluntary to avoid overburdening participants, thereby improving mental health outcomes and maintaining retention rates. To ensure sufficient statistical power, we increased the number of low-risk pregnant participants from 80 to 146, matching the high-risk group (March 2024). This adjustment helps balance the groups since risk status is determined only after enrollment and testing. The change from focusing our primary outcome on two measures: self-ratings of depressive symptoms and clinician-rated PPD to a single measure: clinician-determined PPD (yes/no) is currently (June 2024) under ethical review. While still assessing depressive symptom severity via self-report during the first 6 weeks postpartum, this change aims to provide a more straightforward and valid result, crucial for informing clinical practice. It also reduces the number of multiple comparisons and improves our ability to detect meaningful effects.

The results of this study will have implications for future early prophylactic interventions for pregnant women at heightened risk of PPD. Specifically, if the PACT intervention reduces the incidence of PPD, it can become a short, feasible, non-invasive prophylactic strategy for pregnant women at risk of PPD, with positive mental health implications for these women and their infants. The findings will also provide novel scientific insights into the role of affective cognitive processes for PPD risk and into how targeting these cognitive biases through advanced and tailored training may improve mental health outcomes.

### Supplementary Information

Additional file 1: Table S1. SPIRIT checklist.

Additional file 2: PRS DRAFT Receipt (Working Version).

## Data Availability

The de-identified data can be shared with other researchers upon reasonable request, pending approval by the Data Protection Agency.
