# Supplementary material for: Prenatal affective cognitive training to reduce the risk of postpartum depression (PACT): study protocol for a randomized controlled trial
Source: Trials. 2024 Jul 15;25:478. doi: 10.1186/s13063-024-08316-1 (PMC11247870; doi:10.1186/s13063-024-08316-1)
Supplement: Supplementary file 2 — Additional file 2: PRS DRAFT Receipt (Working Version). [file 13063_2024_8316_MOESM2_ESM.pdf]

ClinicalTrials.gov PRS DRAFT Receipt (Working Version)
Last Update: 07/03/2024 11:04

ClinicalTrials.gov ID: NCT06046456

Study Identification

Unique Protocol ID: R7-A182

Brief Title: Prenatal Affective Cognitive Training to Reduce the Risk of Postpartum Depression (PACT)

Official Title: Prenatal Affective Cognitive Training to Reduce the Risk of Postpartum Depression (PACT): Study Protocol for a Randomized Controlled Trial

Secondary IDs:

Study Status

Record Verification: August 2023

Overall Status: Recruiting

Study Start: January 10, 2023 [Actual]

Primary Completion: February 2025 [Anticipated]

Study Completion: July 2025 [Anticipated]

Sponsor/Collaborators

Sponsor: Mental Health Services in the Capital Region, Denmark

Responsible Party: Sponsor

Collaborators: University of Copenhagen

Oversight

U.S. FDA-regulated Drug: No

U.S. FDA-regulated Device: No

U.S. FDA IND/IDE: No

Human Subjects Review: Board Status: Approved
Approval Number: H-22007428
Board Name: Research Ethics Committee
Board Affiliation: Capital Region of Denmark
Phone: +45 38 66 63 95
Email: vek@regionh.dk
Address:

Borgervænget 3, stuen
2100 København Ø
Denmark

Data Monitoring: No

FDA Regulated Intervention: No

Study Description

Brief Summary: Many pregnant women face the risk of experiencing depression after giving birth, especially if they've previously dealt with depression.

The goal of this study, is to test if our newly developed Prenatal Affective Cognitive Training (PACT) intervention, can decrease the likelihood of post partum depression. In the study, 226 pregnant women, some of whom are considered high risk due to past mental illness or psycho-social risk factors, will participate. The high-risk women will be divided into two groups. One group will undergo the PACT training, which involves computer and virtual reality exercises spanning five weeks, designed to improve mood and emotional regulation. The other group will receive usual care. The study also involves a low-risk group (n=146), although these individuals are not part of the intervention trial but are merely followed up with the same assessments for background comparisons.The main aim is to observe whether the women who undergo PACT intervention are less prone to depression after childbirth compared to those who do not.

This study has potential to offer a simple, non-invasive method to bolster mental health in expectant mothers, which could also positively impact their infants.

Detailed Description: NOTE : Detailed Description has not been entered.

Conditions

Conditions: Postpartum Depression
Emotional Regulation
Cognitive Function 1, Social
Pregnancy Related
Mother-Child Relations

Keywords:

Study Design

Study Type: Interventional

Primary Purpose: Prevention

Study Phase: N/A

Interventional Study Model: Parallel Assignment

Number of Arms: 2

Masking: Single (Outcomes Assessor)

Allocation: Randomized

Enrollment: 146 [Anticipated]

Arms and Interventions

Arms	Assigned Interventions
Experimental: PACT	Prenatal Affective CogntiiveTraining

Arms	Assigned Interventions
Receive the intervention	A psychological intervention using computer- and virtual reality based exercises to modify negative cognitive bias and improve emotion regulation
No Intervention: CAU<br>Receive care as usual, i.e., the standard care provided to pregnant women without any additional experimental interventions. It typically involves routine prenatal care, which may include regular check-ups, medical assessments, and general support from healthcare professionals	

NOTE : An Arm/Group Title this short may not be sufficiently descriptive, especially for later use in results.

NOTE : An Arm/Group Title this short may not be sufficiently descriptive, especially for later use in results.

Outcome Measures

Primary Outcome Measure:

1. The incidence of PPD during the first six months after birth, assessed with the Present State Examination (PSE), which is a part of Schedules for Clinical Assessment in Neuropsychiatry (SCAN)
   The primary outcome is the difference between mothers receiving PACT versus CAU in the incidence of PPD during the first six months after birth, assessed by a clinician blind to group status with the Present State Examination (PSE), which is a part of Schedules for Clinical Assessment in Neuropsychiatry (SCAN)

   [Time Frame: Six months after birth]

Secondary Outcome Measure:

2. Change in negative cognitive bias
   Differences between participants receiving PACT and CAU in the change in negatively biased cognitive processing of infant stimuli from baseline to follow-up during pregnancy (T1) as measured with computerized testing

   [Time Frame: In third trimester of pregnancy]

3. Self-rated depressive symptoms
   Differences between participants receiving PACT and CAU in severity of depressive symptoms during the first six weeks after birth measured with the Edinburgh Postpartum Depression Scale

   [Time Frame: Within six weeks after birth]

4. Self-rated parental stress
   Self-rated parental stress with the Parental Stress Scale at six months after birth.

   [Time Frame: Six months after birth]

Other Pre-specified Outcome Measures:

5. Infant development
   Tertiary outcome involve assessing differences in infant development as measured with the Bayley Scales version 4 between the PACT and CAU groups

   [Time Frame: Eight - eighteen months after birth]

6. Mother-child interaction
   Tertiary outcome involve assessing differences in mother-child interaction measures (e.g., sensitivity, intrusiveness, dyadic reciprocity and neural synchrony) between the PACT and CAU groups as measured with behavioural coding (Coding Interactive Behaviour) and fNIRS brain scan.

   [Time Frame: Eight - eighteen months after birth]

7. Change in affective cognition
   Tertiary outcome involve assessing differences in the changes in facial expressions and visual attention towards infant stimuli (from T0 to T1 during pregnancy) between the PACT and CAU groups, as measured with computerized testing

[Time Frame: Third trimester of pregnancy]

Eligibility

Minimum Age: 18 Years

Maximum Age: 50 Years

Sex: Female

Gender Based:

Accepts Healthy Volunteers: Yes

Criteria: Inclusion Criteria for General Pregnant Population:

- Second or third trimester pregnancy.
- Age ≥ 18 years.
- Ability to speak and read Danish.

Inclusion Criteria for High-Risk Pregnant Group:

- Either:
- Negative cognitive bias in emotional reactivity to infant distress (cut-off > 96 on a scale from 0-100).
- High-risk status according to the Antenatal Risk Questionnaire (ANRQ) which means:
- Personal history of severe mental illness.
- Experienced childhood emotional, physical, or sexual abuse.
- Total score on psycho-social risk factors is above the cut-off (> 23).

Inclusion Criteria for Low-Risk Pregnant Group:

- Absence of a personal or family history of mental illness.
- Absence of negative bias.
- Three or fewer of the additional risk factors mentioned above.

General Exclusion Criteria:

- Schizophrenia.
- Current substance use disorder.
- Score of 9 or more on the Hamilton Depression Rating Scale-6 items (HDRS-6), indicating moderate depression.
- Pregnant women diagnosed with a personality disorder (note: they will still be included at baseline and follow-up after birth but will be excluded from randomization).

Contacts/Locations

Central Contact Person: Anne J Bjertrup, PhD
Telephone: 27134839
Email: anne.juul.bjertrup@regionh.dk

Central Contact Backup: Anne Bjertrup
Telephone: 27134839
Email: anne_bjertrup@hotmail.com

Study Officials: Kamill W Miskowiak, DMsc
Study Principal Investigator
Mental Health Services in the Capital Region of Denmark

Locations: **Denmark**

Mental Health Services in The Capital Region of Denmark
[Recruiting]
Copenhagen, Denmark, 2000
Contact: Anne J Bjertrup, PhD 27134839 anne.juul.bjertrup@regionh.dk

IPDSharing

Plan to Share IPD: No

References

Citations:

Links:

Available IPD/Information:

U.S. National Library of Medicine | U.S. National Institutes of Health | U.S. Department of Health & Human Services
